# Infective Endocarditis in Perceval Sutureless Valves: Incidence, Diagnostic Challenges, and Management: An Expert Opinion Review

**DOI:** 10.3390/diagnostics16060891

**Published:** 2026-03-17

**Authors:** Pau Rello, Lluís Admella Severiano, Arwa Mehmood Wahid, Javier Iglesias-Varea, Joan Roig-Sanchis, Remedios Ríos Barrera, Cristina Kirkegaard-Biosca, Carlota María Vigil-Escalera López, Nuria Vallejo-Camazón, María Nazarena Pizzi, Albert Roque, Nuria Fernández-Hidalgo

**Affiliations:** 1 Department of Medicine, Universitat Autònoma de Barcelona, 08035 Barcelona, Spain; pau.rello@vallhebron.cat (P.R.); lluis.admella@vallhebron.cat (L.A.S.); arwa.mehmood@vallhebron.cat (A.M.W.); nuria.vallejo@vallhebron.cat (N.V.-C.); marianazarena.pizzi@vallhebron.cat (M.N.P.); alberto.roque.idi@gencat.cat (A.R.); 2 Cardiology Department, Hospital Universitari Vall d’Hebron, 08035 Barcelona, Spain; 3 Vall d’Hebron Institut de Recerca (VHIR), 08035 Barcelona, Spain; 4 Infectious Diseases Department, Hospital Universitari Vall d’Hebron, 08035 Barcelona, Spain; javier.iglesias@vallhebron.cat (J.I.-V.); joan.roig@vallhebron.cat (J.R.-S.); cristina.kirkegaard@vallhebron.cat (C.K.-B.); 5 Cardiac Surgery Department, Hospital Universitari Vall d’Hebron, 08035 Barcelona, Spain; remedios.rios@vallhebron.cat (R.R.B.); carlotamaria.vigilescalera@vallhebron.cat (C.M.V.-E.L.); 6 Nuclear Cardiology Unit, Department of Nuclear Medicine, Hospital Universitari Vall d’Hebron, 08035 Barcelona, Spain; 7 CIBERCV—Centro de Investigación Biomédica en Red de Enfermedades Cardiovasculares, Instituto de Salud Carlos III, 28029 Madrid, Spain; 8 Radiology Department, Hospital Universitari Vall d’Hebron, 08035 Barcelona, Spain; 9 Institut de Diagnòstic per la Imatge (IDI), 08035 Barcelona, Spain; 10 CIBERINFEC—Centro de Investigación Biomédica en Red de Enfermedades Infecciosas, Instituto de Salud Carlos III, 28029 Madrid, Spain

**Keywords:** infective endocarditis, perceval, diagnosis, management, prognosis

## Abstract

Sutureless aortic bioprostheses have become an established alternative for surgical aortic valve replacement, particularly in elderly and high-risk patients. The Perceval (Livanova) valve, the most widely studied sutureless device, offers favorable hemodynamic performance and reduced operative times but introduces specific challenges when prosthetic valve endocarditis (PVE) occurs. Although the incidence of Perceval PVE is low and comparable to that of conventional bioprostheses, this complication is associated with substantial morbidity and mortality. Diagnosis is often complex due to acoustic shadowing on echocardiography, making multimodality imaging with transesophageal echocardiography, cardiac computed tomography, and [18F]-FDG PET/CT essential. Microbiological profiles resemble those of other biological prostheses, but perivalvular extension and early mechanical instability are frequent. Management follows general PVE principles but often requires early surgical intervention because of the valve’s reliance on radial fixation. This review summarizes current evidence on epidemiology, microbiology, diagnostic strategies, treatment, and prognosis of endocarditis involving the Perceval valve, and identifies areas for future research.

## 1. Introduction

Sutureless aortic bioprosthetic valves have emerged as an important innovation in cardiac surgery, aiming to simplify implantation and reduce operative times while maintaining the hemodynamic advantages of conventional bioprostheses [1]. Over the past decade, these devices have become an established option for surgical aortic valve replacement (SAVR), particularly in elderly or high-risk patients with severe aortic stenosis. The Perceval valve was the first sutureless prosthesis to gain widespread clinical use and remains the most extensively studied device in this category [1].

The concept of a sutureless valve seeks to bridge the gap between conventional SAVR and transcatheter aortic valve implantation (TAVI). By eliminating or minimizing the need for annular sutures, sutureless valves allow for shorter cardiopulmonary bypass (CPB) and aortic cross-clamp times, potentially reducing perioperative morbidity and facilitating minimally invasive surgical approaches [2]. This technical evolution is especially advantageous in elderly patients, those with multiple comorbidities, or in redo surgeries where annular calcification and tissue fragility make traditional suturing challenging [1,2,3].

The Perceval valve consists of a self-expanding stent frame onto which bovine pericardial leaflets are mounted [2]. Its design allows radial anchoring within the native aortic annulus after decalcification, and its self-expanding properties ensure adequate sealing and positioning without the need for sutures. The device is available in multiple sizes to accommodate a wide range of annular diameters and is deployed under direct visualization using a simple release mechanism. Clinical studies and registry data have demonstrated favourable outcomes with the Perceval valve, including low transvalvular gradients, large effective orifice areas, and acceptable rates of structural valve deterioration over mid-term follow-up [1,2,3].

Despite these advantages, the increasing adoption of sutureless valves has raised new clinical and pathophysiological questions. Among them, prosthetic valve endocarditis (PVE) represents one of the most serious and potentially devastating complications [1]. Its diagnosis and management may pose unique challenges due to the prosthesis’ metallic frame, lack of a sewing ring, and its radiological characteristics on imaging modalities such as echocardiography. Endocarditis on a Perceval valve can be particularly challenging to diagnose due to acoustic shadowing and artifacts on echocardiography, which may obscure vegetations or abscesses.

The mechanisms that predispose to infection in sutureless valves may differ in part from those of traditional prostheses. Moreover, because these valves are often implanted in patients with advanced age and multiple comorbidities, the host-related risk of infection remains significant [4].

Given these complexities, a comprehensive understanding of the epidemiology, microbiology, imaging features, and surgical management of endocarditis on Perceval valves is essential. This review aims to summarize current knowledge regarding the design and technical aspects of sutureless valves, the epidemiology and microbiology of PVE in this context, the diagnostic approach with advanced imaging, and the therapeutic strategies available, and to identify areas for future research.

## 2. Design, Indications and Surgical Characteristics of the Perceval Sutureless Valve

### 2.1. Design

The Perceval sutureless valve is composed of two main components.

First, the tissue component, made of bovine pericardium, undergoes phospholipid reduction using ethanol and 1,2-octanediol, followed by taurine treatment to neutralize free aldehyde residues, and is finally stabilized in a buffered glutaraldehyde solution [5].

Second, the metallic component is a self-expanding nitinol stent coated with Carbofilm to enhance biocompatibility. This structure both supports the prosthesis (the tissue component) and secures it in position. The stent anchors the valve through two cylindrical segments: a proximal segment positioned at the patient’s aortic annulus and a distal segment fitting into the sinotubular junction. These two rings are connected by three straight posts with mid-portion tapering and by six sinusoidal posts designed to conform to the sinuses of Valsalva. The stent structure also renders the prosthesis radiopaque [5].

The tissue component is sutured to the metallic scaffold at the straight posts and covers the lower ring. The nadir of each leaflet corresponds to the midpoint of the lower ring, whereas the free margin aligns with the upper portion of the tapered posts [5].

### 2.2. Indications

As with all bioprosthetic valves, its primary indication—according to international convention—is in patients over 60 years of age [6], as well as those with contraindications to anticoagulation or with reduced life expectancy due to comorbidities.

The prosthesis is designed for AVR in cases of aortic valve disease—stenosis or mixed lesions—where annular rigidity facilitates prosthesis anchoring, like TAVI systems. However, indications have progressively expanded, and Perceval implantation has been reported in pure aortic regurgitation [7] and in cases of failed homografts or degenerated bioprostheses [8].

Certain features make it particularly advantageous in specific situations:

- Minimally invasive surgery [9,10,11]: Because the prosthesis can be folded, implantation through smaller incisions is easier, and visualization of correct positioning is improved both in conventional and minimally invasive approaches, whether via ministernotomy or right lateral minithoracotomy.
- Patients with small annuli [12]: Since the inner diameter of the prosthesis is only 1 mm smaller than that of the stent frame, the effective orifice area is larger compared with sutured bioprostheses, reducing patient–prosthesis mismatch and often avoiding more complex aortic root-enlargement procedures.
- Patients requiring reduced cross-clamp and cardiopulmonary bypass times: Multiple studies [2] show that Perceval™ implantation results in shorter ischemic and bypass times compared with sutured prostheses, making it especially valuable in high-risk patients or in those undergoing concomitant procedures, where reduced times help decrease morbidity and mortality [13,14].

Certain contraindications listed in the technical specifications should also be considered, although many have become relative over time. A classic contraindication is a bicuspid aortic valve; however, several published series have reported successful Perceval implantation in this setting [15,16]. We consider it truly contraindicated only in bicuspid valves with two sinuses of Valsalva. Another contraindication is the performance of concomitant procedures such as multiple valve repair/replacement or coronary revascularization. In multiple valve procedures, Perceval should be implanted last to avoid valve displacement; in the case of proximal coronary anastomoses, these should be performed during cardiac arrest before aortic unclamping [17]. A further contraindication is a dilated ascending aorta when the annulus-to-sinotubular junction ratio exceeds 1.3, as this compromises stent support at the sinotubular level [5]; however, this can be addressed through concomitant ascending aortic replacement. Finally, metal allergy to the materials composing the stent represents an absolute contraindication to Perceval implantation [5].

### 2.3. Surgical Technical Characteristics

Perceval implantation differs from sutured prosthesis placement in several specific aspects [18,19]:

- Aortotomy: A transverse aortotomy—rather than a classical italic-S incision—should be performed 3 cm above the valvular plane to avoid interference with the stent during closure. The periaortic fat band serves as the reference landmark.
- Annular decalcification: Decalcification must ensure a homogeneous surface for prosthesis seating, removing irregularities at the annular, subannular, and supra-annular levels, but without excessive aggressiveness to avoid compromising annular integrity.
- Sizing: The sizing technique has evolved to reduce the initially high pacemaker implantation rates. Currently, prosthesis size should correspond to the largest size that allows a snug passage of the white-barrel sizer through the native annulus. Undersizing may lead to paravalvular leak or migration, whereas oversizing increases the risk of conduction disturbances requiring pacemaker implantation and produces higher postoperative gradients.
- Implantation: Three guiding sutures are placed at the nadirs of the sinuses at 120º intervals. The sutures should enter 2 mm below and exit 2 mm above the annular plane, ensuring proper seating of the prosthesis. After threading the guiding sutures through the prosthesis eyelets, deployment must be performed perpendicular to the annulus while maintaining even tension across the three sutures. Although manual steady traction is standard, the use of snuggers during release (Snugger method) has also been described [20].
- Post-dilatation: Once correct positioning is verified, the prosthesis is dilated at 4 atmospheres for 30 s while irrigating with warm saline.
- Aortotomy closure: Care must be taken to avoid inadvertently entangling the stent frame in the closure sutures.
- Postoperative handling: Gentle manipulation of the heart is essential to prevent prosthesis dislocation or migration. Intraoperative echocardiographic assessment is mandatory to confirm proper function.

## 3. Epidemiology

The incidence of PVE in patients who have undergone AVR with the Perceval valve has been reported in meta-analyses, clinical trials and observational cohorts. Reported incidence rates range between 0.4% and 0.6% per patient-year. Reported cumulative incidence across studies ranges from 0.8% to 6.6% (Table 1) [21,22,23,24,25]. This variability reflects substantial differences in study design—particularly the scarcity of prospective trials—heterogeneous follow-up durations—mean follow-up from 0.9 to 7.03 years [21,26]—differences in baseline characteristics of included populations and, possibly, temporal trends reflecting increasing operator experience and shifts in PVE epidemiology.

A 2024 meta-analysis including retrospective and prospective studies evaluating up to 5-year outcomes of AVR with Perceval valves with or without concomitant procedures reported freedom from infective endocarditis (IE) ranging from 90.7% to 99% [27]. Another meta-analysis of mid-term outcomes found a PVE incidence of 1.6% after a mean follow-up of 4.1 years, with 1.1% of patients requiring Perceval explantation due to IE [28]. Combined data from three multicenter prospective clinical trials—Pilot, Pivotal and CAVALIER—showed a PVE incidence of 1.9% [29], with similar rates reported among patients who underwent AVR with concomitant procedures [17]. Comparable rates of late PVE were observed in a prospective cohort undergoing minimally invasive AVR [11]. In the context of reoperative AVR, a prospective cohort study reported an incidence rate of 0.4% per patient-year [30]. Additionally, a registry-based study found no cases of very early IE (within the first month of AVR) in patients with bicuspid aortic valves treated with sutureless AVR [16].

A detailed description of patient characteristics in Perceval Valve PVE (PPVE) is lacking. A retrospective cohort with detailed reporting noted a mean age of 72 (standard deviation 7.22), a predominance of male patients (85.7%) and a mean EuroSCORE II score of 12.34% (standard deviation 7.4), partially driven by frequent comorbidities such as cardiovascular risk factors (with arterial hypertension and dyslipidemia occurring in 85% of patients), peripheral arterial disease and chronic renal insufficiency [1]. Case reports similarly describe patients in their eighth decade of life affected by chronic cardiovascular comorbidity [31,32].

Although patient profiles are available, no study has identified specific risk factors for PPVE, whether attributable to patient comorbidity, procedural features, or periprocedural events. The risk of IE recurrence after IE requiring AVR with Perceval valves has not been reported.

PPVE remains the most frequent reason for Perceval valve replacement [28,33,34]. Reported operability of PPVE patients ranges from 79% to 100%, mirroring the proportion of those who finally underwent surgical treatment for PPVE, possibly due to the high frequency of indications for surgery [1,35]. However, even if IE undoubtedly contributes to the elevated operative risk, no study has defined the determinants for inoperability in PPVE.

The timing of PPVE remains poorly defined due to the short and heterogeneous follow-up across available cohorts and the limited number of events. A predominance of early PVE (within the first year after AVR) over very early or late PVE (beyond the first year) has been reported in clinical trials [29] and in both prospective observational [11] and retrospective cohorts [1]. In a single-arm prospective trial, the overall risk of PPVE was 1.4%, with most cases (8 out of 9) occurring between the first and the sixth postoperative months [24]. A theoretically relevant distinction between PPVE and PVE involving conventional bioprosthetic valves is the potential for delayed diagnosis due to the absence of perivalvular leak in PPVE, potentially related to the self-expanding radial forces of the Perceval valve [31,32]. However, no study has directly compared the time distribution of PPVE with that of PVE affecting conventional bioprostheses.

No direct comparative studies evaluating the incidence or clinical and radiological manifestations of PPVE relative to those of PVE affecting conventional aortic bioprotheses exist. Available evidence is indirect and sparse. A case report of PPVE complicated with aortic abscess and aortic root extension describes a pattern of infection extension—particularly, a tendency to extend toward the left ventricular outflow tract—but these observations are still insufficient to define an epidemiological pattern [31].

**Table 1:** Relevant observational studies and clinical trials on the epidemiology of infective endocarditis following Perceval valve implantation.

First Author	Study Design	Study Period	Perceval Population (*n*)	Perceval IE Cases (*n*)	Perceval IE Incidence Rate (Per 100 Patient-Years)	Perceval IE Cumulative Incidence (%)	Follow-Up Mean or Median Duration	Perceval IE Mortality (%)
Berastegui [1]	Retrospective cohort study; single-center	2015–2020	670	14	NR	2.1	23 months	14 (in-hospital), 41 (during follow-up)
Berastegui [33]	Prospective cohort study; multicentric	2013–2016	448	2	NR	0.45	1 year	NR
Di Bacco [25]	Retrospective cohort study; multicentric	2012–2015	518	8	0.6	NR	3.1 years	NR
Dokollari [22]	Systematic review and meta-analysis	-	12,714	NR	NR	1.6–6.6	-	NR
Flameng [35]	Retrospective cohort study; single-center	2007–2009	32	1	NR	3.13	15.8 months	NR
Glauber [11]	Prospective cohort study; multicentric	2011–2018	480	4	0.4	NR	2.4 years	NR
Jolliffe [28]	Systematic review and meta-analysis	-	3196	50	NR	1.6	-	NR
Lamberigts [26]	Retrospective cohort study; single-center	2007–2019	784	13	0.46	1.7	7.03 years	NR
Rubino [21]	Retrospective cohort study; multicentric	2007–2013	314	NR	NR	0.80	0.9 years	NR
Suri [23]	Single-arm clinical trial; multicentric	2013–2025	300	5	NR	1.7	1 year	NR

NR: not reported.

## 4. Microbiology

Information regarding the association between specific microorganisms and IE in the Perceval prosthesis is scarce. Most available data are derived from systematic reviews evaluating mid- and long-term outcomes of the Perceval valve and other sutureless bioprosthetic valves. Overall, it seems that the microbiological profile is similar to that reported for other biological prostheses, with streptococci, enterococci, and coagulase-negative staphylococci being the most frequently isolated organisms. The few series focusing only on Perceval endocarditis include fewer than 15 patients and provide limited microbiological data [27,28,31,34,36]. The incidence of both early and late PVE in the Perceval prosthesis was similar to that reported in the literature for SAVR [28], with cases of IE occurring from a few months up to 3 years after implantation [37,38]. There is just one study that focuses on the microbiological data for Perceval IE, which found that the most frequently isolated microorganism was *Staphylococcus epidermidis* (7 out of 14 patients), and most cases (78.6%) were early PVE with an interval of more than 3 months after surgery [1]. Another work based on a case report describes a case of IE caused by methicillin-resistant *Staphylococcus aureus* 23 months after implantation [31].

## 5. Clinical Presentation and Diagnosis

The clinical behaviour of PVE in sutureless devices, specifically the Perceval prosthesis, is fundamentally dictated by its unique anchoring mechanism. This design necessitates a specific pathophysiological understanding when infection occurs.

Current clinical evidence identifies a significant incidence of early-onset PVE, typically detected from the first month onwards following implantation. These cases are frequently associated with perioperative bacteraemia or nosocomial pathogens acquired during the initial surgical hospitalization. Conversely, late-onset PVE—defined as occurring more than one year after the procedure—follows a more indolent, subacute course. This late-stage infection often involves community-acquired pathogens and mimics the clinical progression observed in traditional stented bioprosthetic valves [34,39,40].

### 5.1. Clinical Presentation and Symptomatology

Fever remains the most consistent clinical hallmark of PVE, documented in over 80% of the patient population. However, a high degree of clinical suspicion is required, as the fever may be absent or suppressed. This is particularly relevant in the Perceval recipient cohort, which often comprises elderly patients with multiple comorbidities or those receiving chronic anti-inflammatory medications.

Beyond pyrexia, the clinical spectrum encompasses non-specific constitutional symptoms such as generalized malaise, fatigue, and anorexia. In more virulent or aggressive presentations, patients may rapidly progress to septic syndrome characterized by hemodynamic instability and a significant rise in acute-phase reactants. The presence of these biomarkers, while non-specific, is a critical component of the diagnostic workup when evaluated alongside the patient’s surgical history [4].

### 5.2. Mechanical Failure and Hemodynamic Dysfunction

A distinctive and critical feature of endocarditis in the Perceval valve is the early compromise of the periprosthetic seal. The infection typically colonizes the interface between the flexible nitinol frame and the native aortic annulus. As the infection progresses, it undermines the radial force and prosthetic apposition, leading to rapidly progressive paravalvular leaks.

From a clinical perspective, this mechanical failure manifests as acute or subacute heart failure. The loss of anchorage results in sudden valvular dysfunction or massive regurgitation. The detection of a de novo cardiac murmur in a patient with a sutureless valve is a clinical emergency that necessitates immediate imaging to exclude prosthetic dehiscence. Furthermore, while classic peripheral stigmata—such as Janeway lesions or splinter hemorrhages—are statistically less common in PVE, their presence provides definitive evidence of systemic involvement [1,37].

### 5.3. Systemic Embolism and Perivalvular Complications

Systemic embolic events are reported in 20% to 30% of cases and usually involve the central nervous system, the spleen, and the kidneys. In many instances, an embolic stroke or acute abdominal pain serves as the initial clinical manifestation of the underlying endocarditis.

The proximity of the nitinol frame to the conduction system also increases the risk of serious local complications. The formation of perivalvular abscesses and pseudoaneurysms is a frequent and severe progression of the disease. These invasive processes often manifest as new-onset conduction disturbances, such as high-grade atrioventricular blocks. Such findings indicate deep tissue destruction and often suggest the involvement of the left ventricular outflow tract, carrying a poor prognosis without urgent surgical intervention [41].

### 5.4. The Multimodal Diagnostic Paradigm

The diagnosis of IE in the Perceval valve must be managed through a multimodal framework established by the 2023 Duke-ISCVID Criteria and the 2023 ESC Guidelines. Central to this approach is the “Endocarditis Team,” a multidisciplinary group tasked with synthesizing complex clinical, microbiological, and imaging data.

Microbiological identification remains a cornerstone of the diagnosis. It is mandatory to obtain at least three sets of blood cultures prior to commencing antimicrobial therapy. The identification of typical microorganisms, such as *Staphylococcus* or *Enterococcus* species, provides the microbiological major criterion required for a definitive diagnosis [42].

## 6. Imaging Diagnosis of Infective Endocarditis in the Perceval Prosthesis

The specific characteristics of these sutureless prostheses suggest that a multimodality imaging (MMI) approach to achieving a definitive diagnosis of infection in Perceval valves may be required more often than in other surgically (sutured) implanted prostheses, and may be more comparable to TAVI-IE.

### 6.1. Transthoracic Echocardiography (TTE) and Transesophageal Echocardiography (TEE)

Transthoracic echocardiography (TTE) and transesophageal echocardiography (TEE) are the first-line imaging modalities for suspected PVE in sutureless Perceval prosthesis patients, but diagnosis in this context is challenging.

There are no studies specifically addressing the limitations of echocardiography in Perceval PVE. In similar scenarios, such as transcatheter aortic valve implantation (TAVI), the combined sensitivity of TTE and TEE for the diagnosis of TAVI-IE is about 67%, compared with 73% in patients with surgically implanted prosthetic valves [43].

The design of the Perceval valve creates specific diagnostic pitfalls on TEE imaging (similar to those observed with TAVI prostheses) and differs from the diagnostic findings in other forms of PVE.

Some of these findings are specifically detailed below:

- Vegetations: Mobile, irregular echo-bright masses on the prosthetic leaflets or at the sewing ring/interface with the aorta. The metal stent creates shadows and artifacts that can obscure vegetations. Conversely, valve “struts” and folds of pericardial leaflet tissue can be mistaken for vegetations.
- Leaflet thickening and obstructive patterns: Thickened bioprosthetic leaflets and increased transvalvular Doppler gradients or reduced valve opening (possibly due to vegetative infiltration or thrombus) can be observed. These changes, while nonspecific, may raise suspicion in the appropriate clinical context.
- Paravalvular complications/abscesses: A thickened, non-homogeneous perivalvular area with echolucent zones. In the Perceval valve, it often manifests as a space between the stent and the aortic wall. In some series, periannular thickening (<10 mm) on TEE is not diagnostic of abscess, and periannular extension is clearly larger when cardiac computed tomography is performed [1].
- Perivalvular leak/aortic regurgitation: Due to the special design of the Perceval prosthesis, which anchors the valve to the aortic annulus by means of the radial force of the nitinol stent alone, aortic regurgitation due to perivalvular leak is not present in the early stages of infection. The lack of periprosthetic leak could delay the diagnosis, especially if the images of periannular involvement are not diagnostic of abscess in TEE [1]. In advanced stages of the infection, anatomical dehiscence with stent “rocking” and secondary severe paravalvular aortic regurgitation may be observed, even on TTE.

Given the limitations of echocardiography in this context, a multi-modality approach with advanced imaging techniques such as cardiac CT and 18F-fluorodeoxyglucose positron emission tomography/computed tomography ([18F]FDG-PET/CT) is recommended. These modalities can facilitate early diagnosis when there is high clinical suspicion, allowing better evaluation of periannular anatomical involvement, even when TEE is negative or non-conclusive or in patients with contraindications to TEE evaluation.

### 6.2. Cardiac CT and [18F] FDG PET/CT in the Diagnosis of Perceval Valve Endocarditis

A multimodality work-up may include cardiac computed tomography (CT) and 18F-fluorodeoxyglucose positron emission tomography/computed tomography ([18F]FDG-PET/CT), which have consolidated their role in IE. Nevertheless, there are no specific recommendations in the guidelines regarding the use of cardiac CT or [18F]FDG-PET/CT depending on the subtype of prosthetic valve. Accordingly, the work-up in suspected Perceval valve infection should follow the general indications for prosthetic valve endocarditis (PVE) [4,42]. Moreover, the diagnostic accuracy of CT and/or PET/CT has been evaluated in the setting of suspected PVE in general series in which the specific prosthetic valve models were not well reported and may or may not have included sutureless valves. Therefore, until specific data become available, it is reasonable to extrapolate to Perceval valves the general diagnostic performance and application of the general guideline-based indications established for PVE.

#### 6.2.1. CARDIAC CT

Cardiac CT provides high spatial resolution for the anatomical and functional assessment of prosthetic heart valves and has proven to be useful in the diagnosis of infective endocarditis and the detection of its complications [44]. In particular, cardiac CT has shown higher sensitivity for the detection of periprosthetic complications, such as abscesses and pseudoaneurysms, when compared to transesophageal echocardiography (TEE) (87% for CT vs. 69% for TEE) [45]. This advantage is especially useful in the suspicion of endocarditis involving Perceval prostheses, as these valves frequently develop periprosthetic abscesses, sometimes even in the absence of clearly identifiable vegetations. On CT, abscesses appear as periprosthetic soft-tissue thickening with variable extent. If disruption of the aortic wall or other structures occurs, cardiac CT also allows the detection of pseudoaneurysms. Although CT is not superior to echocardiography, it can also identify vegetations, which may be located on the leaflets or at different sites along the stent in this type of device. Vegetations may not be easily visualized by echocardiography due to metallic shadowing and artifacts. In addition, CT provides valuable information for surgical planning, including non-invasive assessment of coronary anatomy and atherosclerotic disease if patients require cardiac surgery.

#### 6.2.2. [18F]FDG-PET/CT

[18F]FDG-PET/CT is a hybrid imaging technique that has demonstrated significant diagnostic utility in PVE, allowing the reclassification of a large proportion of cases that initially remain categorized as doubtful or “possible” after clinical and echocardiographic evaluation [46]. The added value of PET/CT in PVE has been confirmed across patient series with mixed prosthetic valve types, or even in cohorts not including sutureless models [47]. In general, a focal, multifocal, or heterogeneous FDG uptake pattern, visualized on the valve stent, is highly suggestive of infection. FDG activity extending into the periaortic space may suggest a possible periprosthetic abscess. When PET/CT is performed as a cardiac-dedicated examination, including cardiac CT within a single acquisition, it provides not only metabolic information but also allows the detection of the associated anatomic lesions (vegetations and periprosthetic complications), thereby significantly improving diagnostic yield.

Furthermore, both CT and [18F]FDG-PET/CT allow extracardiac evaluation for the detection of distant lesions, which are common in the setting of endocarditis. These findings may also fulfill minor criteria useful for reclassification of patients with suspected infective endocarditis and can contribute meaningfully to overall patient management [47].

**Figure 1:**
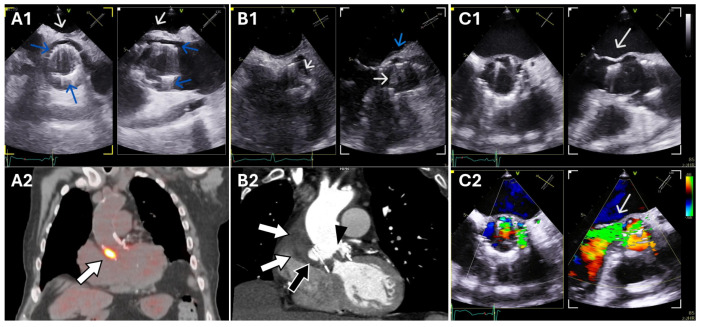
Three cases of PVE in Perceval prosthetic valves. Panel (**A1**). Pseudo-sinus (blue arrows) and non-specific periprosthetic thickening (white arrow). Panel (**A2**). [18F]FDG-PET/CT fused image in the same patient showing intense FDG uptake on the prosthetic valve consistent with infection with periprosthetic involvement. Panel (**B1**). Leaflet thickening suggestive of vegetations (white arrows) and posterior periannular thickening (blue arrow); panel (**B2**). Corresponding cardiac CT confirmed vegetations (arrowhead) associated with periprosthetic and periaortic abscess (white arrows and black arrow). Panels (**C1**,**C2**). Perceval prosthesis dehiscence and severe paravalvular regurgitation (arrow). PVE, prosthetic valve endocarditis; [18F]FDG-PET/CT, 18F-Fluorodeoxyglucose Positron Emission Tomography/Computed Tomography; CT, Computed Tomography.

PET/CT can also identify the portal of entry of the infection of certain microorganisms, such as colonic lesions, which is particularly relevant in infective endocarditis, as eradication of the infectious source may prevent relapse and/or reinfections [47]. Furthermore, when endocarditis is ruled out, PET/CT can suggest an alternative infectious or noninfectious diagnosis. Another important advantage of PET/CT is that, when endocarditis is excluded, it can suggest alternative infectious or noninfectious diagnoses. Figure 1 presents three representative cases assessed by echocardiography and PET/CT.

**Figure 2:**
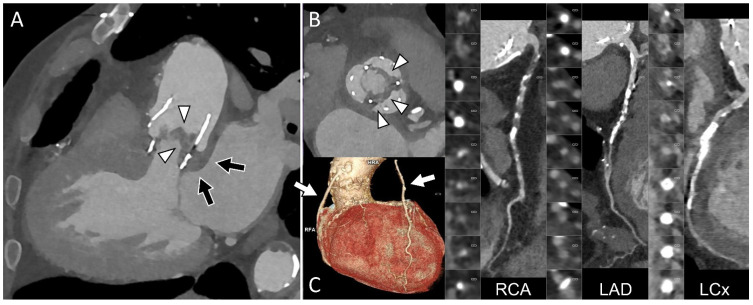
Comprehensive cardiac CT assessment in prosthetic valve endocarditis involving a Perceval sutureless valve. Echocardiography was diagnostic for valvular vegetations, but the presence and extent of perivalvular involvement remained uncertain. Cardiac CT was therefore performed to evaluate periprosthetic complications and to assess coronary anatomy prior to surgery. Three-chamber (**A**) and aortic plane (**B**) views confirm leaflet vegetations (arrowheads) associated with extensive periprosthetic soft-tissue thickening extending to the mitral–aortic fibrosa (black arrows), consistent with a perivalvular abscess ((**A**), black arrows). CT coronary angiography demonstrated severe and diffuse coronary artery disease but patency of two coronary bypass grafts ((**C**), arrows), neither of which was in close proximity to the predicted re-sternotomy plane.

**Figure 3:**
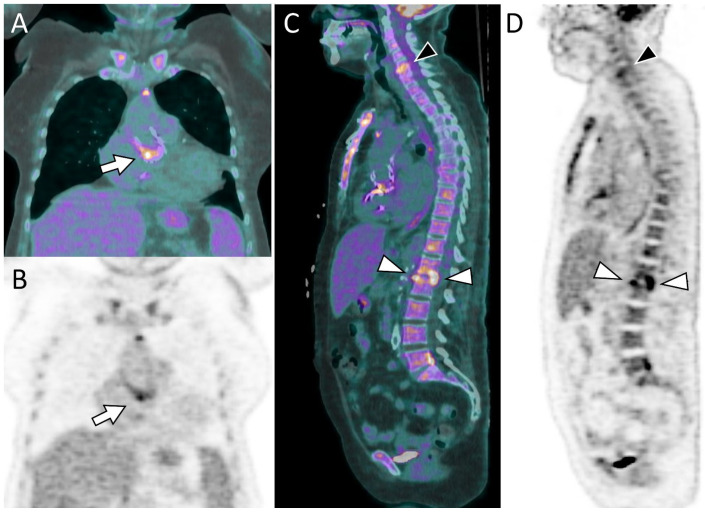
A 76-year-old woman with a Perceval prosthetic aortic valve and positive blood cultures for *Enterococcus faecalis*. Echocardiography revealed a small mobile structure that was inconclusive for vegetation, prompting further evaluation with [^18^F]FDG PET/CT. Fused (**A**) and attenuation-corrected (**B**) coronal images demonstrate focal and intense FDG uptake at the level of the Perceval prosthesis (arrows), consistent with prosthetic valve endocarditis. (**C**,**D**) Whole-body imaging additionally revealed findings consistent with spondylodiscitis at the L1–L2 (white arrowheads) and C5–C6 (black arrowhead) levels.

Since Perceval valve implantation is usually selected for patients at moderate to high surgical risk, particularly elderly patients [48], some individuals with Perceval valve endocarditis may be considered poor candidates for cardiac surgery and managed with long-term antibiotic therapy. In this context, PET/CT findings can support clinical decision-making and may be used to monitor the response to antimicrobial treatment [48]. Figure 2 highlights the importance of a comprehensive CT assessment, whereas Figure 3 presents a case of endocarditis with spondylodiscitis, illustrating the value of PET/CT in a patient with inconclusive echocardiographic findings.

**Figure 4:**
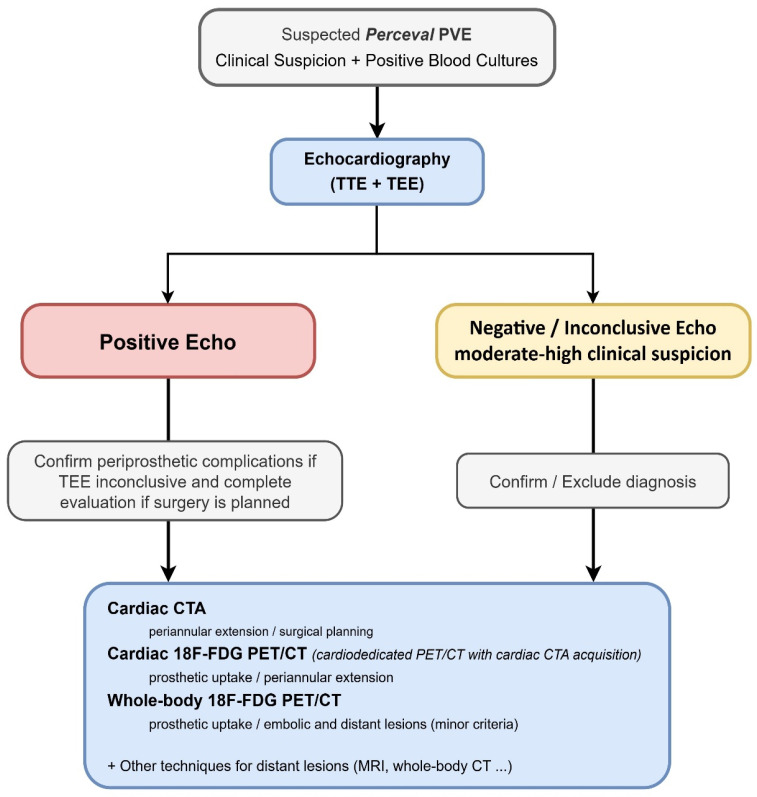
Recommended diagnostic approach for patients with a Perceval Valve and suspected IE.

Based on the information above, a specific diagnostic approach is recommended for patients with a Perceval valve and suspected IE (Figure 4).

## 7. Treatment

### 7.1. Antimicrobial Treatment

Evidence specifically addressing antimicrobial treatment for infective endocarditis involving the Perceval valve is limited, and no valve-specific therapeutic recommendations are currently available. Consequently, antibiotic management follows the general principles applied to patients with prosthetic valve infective endocarditis [4].

Empirical therapy should therefore be guided by the timing of infection onset. In early prosthetic valve endocarditis (<12 months after valve implantation), microorganisms typically associated with healthcare exposure, particularly coagulase-negative Staphylococcus, are predominant. In contrast, in late prosthetic valve endocarditis (≥12 months after implantation), the microbiological spectrum more closely resembles that of native valve endocarditis [49]. Once the causative pathogen has been identified, antimicrobial therapy should be tailored accordingly. In the absence of complications, the recommended duration of antibiotic treatment is at least 6 weeks [1].

Although no alternative antimicrobial regimens can be recommended specifically for Perceval valve endocarditis, several aspects of this patient population warrant particular attention in clinical practice. First, this sutureless bioprosthetic valve, owing to its simplified implantation technique, is preferentially used in older patients and in those with significant comorbidities [1,37], who are more susceptible to antibiotic-related adverse events, including nephrotoxicity, fluid overload, and pharmacological interactions. As a result, careful individualized risk–benefit assessment is essential when selecting antimicrobial therapy. The use of agents such as aminoglycosides or rifamycins requires careful assessment and close monitoring, given their potential for severe toxicity in this patient population. Whenever feasible, antimicrobial regimens should be selected with the goal of preserving efficacy while minimizing treatment-related toxicity.

Second, owing to the valve’s anchoring and deployment mechanism, there is evidence of a higher incidence of perivalvular involvement [1], including abscess formation [31]. As a result, surgical management represents a cornerstone of treatment in these cases. In patients who are not candidates for repeat surgical intervention, standard-duration antibiotic therapy is often insufficient, and prolonged or suppressive antimicrobial treatment should be considered to achieve infection control, with high rates of failure and high mortality associated [50].

### 7.2. Surgical Indications in Perceval Prosthesis Infective Endocarditis

Surgical indications for infective endocarditis (IE) affecting a Perceval sutureless aortic prosthesis follow the principles applied to PVE, as outlined in the 2023 ESC guidelines [4]. Even so, the Perceval does not behave like a conventional sutured bioprosthesis when infection involves the annulus. As a result, patients often reach surgical thresholds earlier, making prompt Heart Team assessment essential.

The classic surgical triggers in PVE (acute prosthetic dysfunction with heart failure, persistent infection, perivalvular complications and significant embolic risk) apply fully to Perceval infections. These indications are supported by recent literature analysing sutureless prostheses and Perceval-specific series [51,52]. In practice, the mechanical vulnerability of this design means that these criteria tend to be met sooner when annular or perivalvular involvement develops.

Prosthesis instability is one of the most decisive reasons to proceed early. Because fixation depends exclusively on radial force, even small areas of annular compromise (such as an early abscess, localized necrosis or a phlegmon) can destabilize the prosthesis and lead to partial or complete dehiscence [1]. This differs from sutured valves, where isolated infected stitches may still allow temporary stability. In Perceval prostheses, loss of support can be abrupt, and imaging signs of instability (rocking, displacement or new paravalvular leakage) should be considered urgent indications for surgery, even in the absence of advanced heart failure.

Acute leaflet dysfunction is another clear trigger for intervention. Infectious destruction, obstructive vegetations or sudden severe regurgitation cannot be managed conservatively in a sutureless system. Repair is not feasible, and significant dysfunction nearly always leads to explantation. Surgical reintervention may be complex due to inflamed or friable annular tissue, as reported in several case series [53]. Early surgery therefore helps prevent further damage and reduces operative difficulty.

Perivalvular complications—abscesses, pseudoaneurysms and fistulas—are also strong indications for early intervention. The lack of a sewing ring offers minimal resistance to the outward spread of infection, which may accelerate the formation of invasive lesions [1,29]. When such complications are detected or suspected, early surgery is advisable, regardless of temporary clinical stability.

Vegetations ≥ 10–15 mm or those showing marked mobility carry a high embolic risk and remain a well-established reason for surgery. Although comparative imaging data are limited, the open-frame design of the Perceval may allow greater mobility of attached vegetations. Observational studies and expert reviews support early intervention in this setting [29,54].

Microbiology significantly influences timing. *Staphylococcus aureus*, fungi and non-HACEK Gram-negative organisms often produce rapid tissue destruction. Given the limited mechanical tolerance of the Perceval prosthesis, these pathogens frequently lead to earlier surgical indication and make prolonged medical therapy alone unreliable [4,55].

The potential role of valve-in-valve transcatheter aortic valve implantation (TAVI) in the setting of active infective endocarditis remains unclear, as evidence is scarce and limited to anecdotal reports. To date, published data consist only of isolated case reports involving conventional bioprosthetic valves, including a recent single case [55], and no studies have specifically addressed its use in Perceval sutureless prostheses. In light of this limited evidence, no therapeutic recommendation can be drawn. That said, in exceptional circumstances—particularly in patients considered unsuitable for surgery—valve-in-valve TAVI could be contemplated as a rescue approach after adequate infection control and thorough multidisciplinary Heart Team discussion. Further clinical experience and dedicated studies are needed to better define its potential role in this context.

### 7.3. Technical Surgical Considerations for Endocarditis on a Perceval Prosthesis

Large series describing Perceval explantation are lacking, and most of the recommended surgical maneuvers have been extrapolated from the extraction of self-expanding transcatheter valves due to their structural similarities [56].

When facing a Perceval prosthesis that must be explanted—whether due to endocarditis or structural degeneration—preoperative imaging is essential to evaluate the degree of endothelialization of the stent frame. However, unlike in transcatheter prostheses, these findings do not guide the height of the aortotomy or the cannulation strategy. In general, explantation of a Perceval prosthesis is performed through a median sternotomy with central cannulation. The site of the aortotomy is usually determined by the location of the previous one.

Once the prosthesis is exposed, two main strategies have been described for its removal. In both techniques, the critical step is the accurate identification of the dissection plane between the prosthesis and the aortic intima [57]. After locating this plane, extraction may proceed using one of the following methods:

#### 7.3.1. X-Shaped Maneuver [32]

This is the technique traditionally used when the prosthesis must be repositioned during the initial implant. Using two forceps placed 180° apart on the upper crown of the stent, opposing traction is applied to create an “X-shaped” motion that detaches the stent posts from the aortic wall, facilitating prosthesis removal. This maneuver is particularly useful in recent implants or in cases with minimal adherence of the stent to the aorta and aortic annulus.

#### 7.3.2. “Lasso” Technique [58]

Originally described for the extraction of self-expanding transcatheter valves, this technique can be adapted, with minor modifications, for Perceval prostheses. Once the dissection plane and the upper crown of the prosthesis are identified, a circumferential suture is passed around the crown and secured with a tourniquet, creating centripetal traction that partially collapses the prosthesis. This controlled folding facilitates traction, mobilization, and meticulous dissection of the prosthesis, ultimately enabling safe removal.

In both techniques, irrigating the prosthesis with cold saline has been reported as beneficial, as it induces contraction of the nitinol stent [59], aiding dissection and reducing the risk of structural complications.

Following prosthesis explantation, the surgical approach depends on the extent of structural damage caused by endocarditis—such as abscesses, pseudoaneurysms, or fistulous tracts—as well as by the extraction maneuvers. The surgical strategy may range from simple aortic valve replacement with a new prosthesis to replacement combined with annular or root repair procedures, or even complete aortic root replacement. The need for associated or more complex procedures arises in up to 50% of cases when removing self-expanding prostheses [59]. 

**Figure 5:**
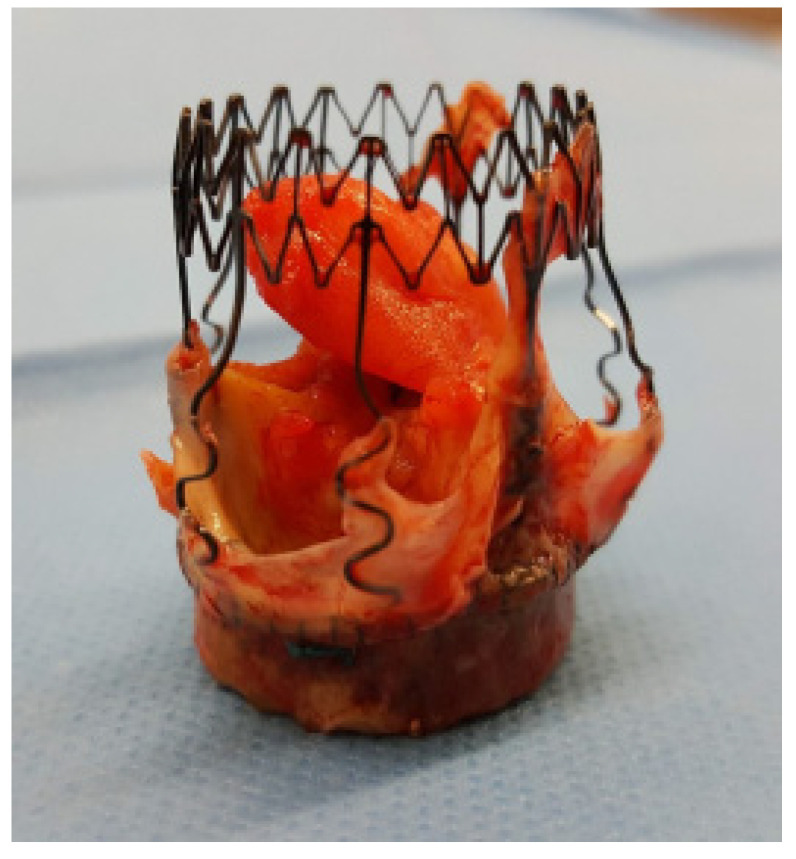
Explanted Perceval prosthesis with a large attached vegetation.

**Figure 6:**
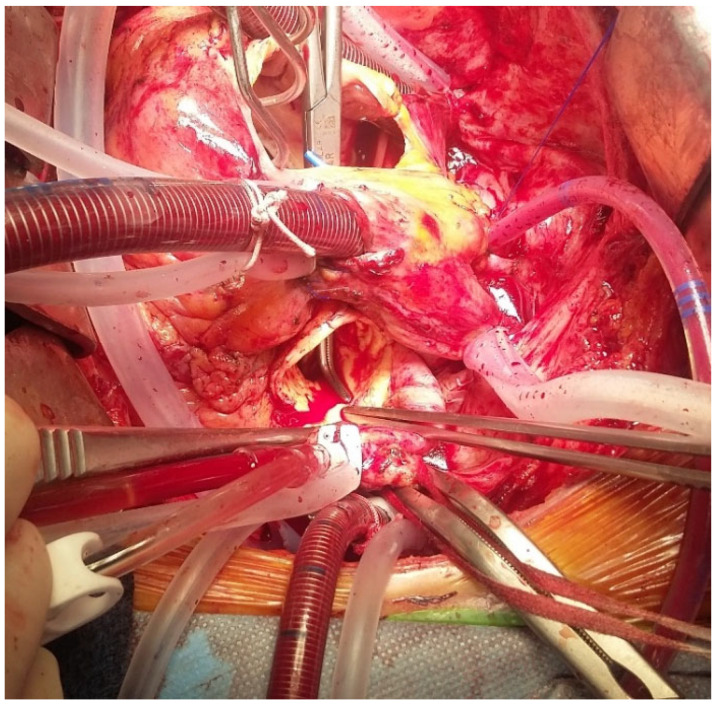
Aortic to right atrium fistula (highlighted by the path of the dissector) in a case of endocarditis on a Perceval prosthesis.

Figure 5 and Figure 6 show an explanted Perceval valve and intraoperative findings of endocarditis involving a Perceval valve.

## 8. Prognosis

### 8.1. Comparative Durability

The clinical landscape of Sutureless Aortic Valve Replacement (Su-AVR) has been significantly shaped by the performance of the Perceval valve. Current evidence suggests that the incidence of PVE in these models is notably low. Long-term follow-up studies have reported freedom from PVE rates as high as 98% at the 8-year mark [40]. However, a critical observation in longitudinal cohorts is that PVE has emerged as the primary indication for late valvular explant. This stands in stark contrast to conventional bioprostheses, as Structural Valve Deterioration (SVD) is remarkably rare—and in many series, practically non-existent—within this specific sutureless model [37,40]. Consequently, while the valve demonstrates exceptional mechanical durability, infection remains the most significant threat to its long-term viability.

### 8.2. Prognostic Determinants and Clinical Gravity

The prognosis for patients developing PPVE is intrinsically severe, mirroring the high-risk profile associated with any prosthetic heart valve infection. Contemporary registries indicate a hospital mortality rate ranging between 25% and 35% [60,61]. This elevated mortality is often a reflection of the patient population typically selected for sutureless valves—often older individuals with higher EuroSCORE II values and multiple comorbidities. Survival outcomes are drastically undermined by the rapid onset of intracardiac complications. Specifically, the development of perivalvular abscesses, acute heart failure, septic shock, or acute kidney injury serves as a primary driver of poor clinical outcomes.

### 8.3. Therapeutic Strategies: The Surgical Imperative

Perioperative mortality for redo interventions in prosthetic endocarditis remains high, often exceeding 25%. Despite this surgical risk, the data overwhelmingly support an aggressive surgical approach when indicated. Large-scale retrospective analyses have demonstrated a striking disparity in outcomes based on management strategy: patients treated with medical therapy alone faced a 34% mortality rate, compared to only 9% among those who underwent surgical intervention [61]. These figures reinforce the critical role of timely surgery, despite the inherent technical complexity of explanting a nitinol-based frame and debriding the aortic root. For those who survive the acute phase, long-term survival rates are comparable to those seen in native valve endocarditis, though patients must be closely monitored for recurrent infection or residual hemodynamic dysfunction [40,61].

### 8.4. Technical Advantages in Redo Scenarios

Notably, the Perceval valve serves not only as a subject of PVE but also as a feasible therapeutic option for its management. In cases of active endocarditis necessitating re-replacement, the device has demonstrated clinical utility as a redo surgical option. Its sutureless design facilitates rapid deployment, which significantly reduces cardiopulmonary bypass and aortic cross-clamp durations—parameters that are critical for survival in hemodynamically unstable patients with high operative risk [40].

Furthermore, its favourable hemodynamic performance and the absence of a conventional sewing ring may allow for improved adaptation to a friable or damaged aortic annulus.

## 9. Future Directions

Endocarditis is a constantly evolving disease, increasingly affecting complex patient populations, many of whom are elderly and have a high burden of comorbidities. At the same time, the incidence of infections involving prosthetic valves, aortic grafts, and cardiac implantable electronic devices continues to rise. As with many other cardiovascular conditions, this heterogeneity of clinical presentations underscores the need for an individualized approach. Despite this, epidemiological studies describing endocarditis according to the type of valve affected remain scarce. In this context, although Perceval prostheses are gaining acceptance and are increasingly used in surgical practice, there are still very few published case series reporting PVE.

Therefore, an individualized approach to PVE is warranted, supported by robust population-based incidence data and clinical registries that take into account the type of prosthesis, both those currently available and those that may become available in the future. Such registries should incorporate detailed clinical, microbiological, imaging, and prognostic information. Additionally, there is a critical need to determine the optimal duration of antimicrobial therapy for patients who have a surgical indication but cannot be operated on, particularly patients with paravalvular involvement. In this scenario, advanced imaging techniques, such as PET-CT, may play a key role in guiding clinical decisions, including the timing of antimicrobial withdrawal. A comprehensive analysis of these factors will help refine not only the diagnostic strategies but also the management and outcomes of these highly complex patients.

## 10. Conclusions

Sutureless aortic bioprostheses, and particularly the Perceval valve, represent a major step forward in the surgical treatment of aortic valve disease. However, as their use expands, clinicians are increasingly faced with complex postoperative complications such as prosthetic valve endocarditis.

Endocarditis involving a Perceval valve remains an uncommon but severe entity associated with high morbidity and mortality. Diagnostic accuracy continues to depend on a multimodality imaging approach, combining echocardiography and nuclear imaging techniques. Management requires a tailored strategy that balances antimicrobial therapy and timely surgical intervention. Surgical explantation of the Perceval prosthesis can be technically demanding due to its self-expanding metallic frame and potential adhesion to the aortic wall.

Future research should focus on refining perioperative infection prevention strategies, understanding the pathophysiological mechanisms of bacterial adherence specific to sutureless devices, and optimizing both medical and surgical management protocols.

In conclusion, while sutureless aortic bioprostheses have broadened the therapeutic options for aortic valve disease, vigilance for infectious complications remains crucial. The multidisciplinary collaboration among cardiologists, cardiac surgeons, infectious disease specialists, and imaging experts is fundamental to improving prognosis and ensuring the continued safe use of these innovative devices.

## Data Availability

The data supporting the findings of this study are not publicly available due to ethical and privacy restrictions.
